# Programmable π-electron quantum matter: from molecular qubits to correlated and topological states

**DOI:** 10.1093/nsr/nwag185

**Published:** 2026-03-31

**Authors:** Lifeng Chi

**Affiliations:** State Key Laboratory of Bioinspired Interfacial Materials Science, Institute of Functional Nano and Soft Materials (FUNSOM), Soochow University, China

Recent advances at the interface of chemistry and condensed matter physics are transforming π-conjugated molecular systems into a versatile platform for quantum materials. The eight papers collected in this Special Topic highlight how chemical design, electron correlation, topology and interfacial effects can be combined to realize emergent quantum states with increasing controllability.

In a Perspective, Ma and Feng discuss carbon-based molecular qubits as a promising route toward ambient quantum technologies [1]. They emphasize that carbon-based open-shell systems, especially nanographene diradicaloids, benefit from weak spin–orbit coupling and reduced hyperfine interactions, enabling relatively long spin coherence time. Importantly, the authors argue that chemical synthesis provides an unparalleled degree of structural tunability, allowing control over spin multiplicity, coupling and environment. They also outline key challenges, including decoherence mechanisms, stability of open-shell systems and scalable integration into devices.

For the Research Articles, several studies demonstrate how distinct quantum phenomena can be engineered in molecular and low-dimensional systems. Zhu, Liu, Chi and Pan report the realization of a Mott insulating state in a two-dimensional metal–organic Kagome framework [2]. By combining scanning tunneling spectroscopy and theoretical calculations, they show that flat bands near the Fermi level split into Hubbard bands due to strong electron–electron interactions, leading to a correlation-driven gap that closes with increasing temperature or carrier doping. This work establishes metal–organic frameworks as a new platform for exploring flat-band correlation physics.

Ma and Wang demonstrate that topological phases can be chemically engineered in single π-conjugated polymer chains [3]. Through precise end-group modification, they create spatially distinct trivial and non-trivial segments within a single polymer, enabling the formation of topological solitons at domain walls. The coexistence of band inversion, zero-energy states and localized vibrational signatures provides compelling evidence of controllable topological excitations at the molecular scale.

Zhang and Du propose a new mechanism for anti-ferromagnetism in hydrogen-bonded organic frameworks [4]. Their theoretical study shows that charge transfer between donor and acceptor molecules arranged on interpenetrating sub-lattices can induce spin polarization, resulting in a nesting-type antiferromagnetic ground state. This mechanism does not rely on traditional magnetic elements, highlighting the potential of purely organic systems for unconventional magnetic order.

Xiang, Torres, Bottari and Jelínek investigate triangulene-fused porphyrins synthesized on Au(111) surfaces [5]. They demonstrate that these hybrid nanographene–porphyrin systems possess intrinsic open-shell character with anti-ferromagnetically coupled spins, but their electronic and magnetic states are strongly influenced by interfacial charge transfer with the substrate. The work highlights the importance of molecule–substrate interactions in determining the magnetic properties of surface-supported systems.

Baryshnikov and Xu explore the tuning of aromaticity in cyclocarbons via heteroatom doping [6]. By introducing sulfur or nitrogen atoms into C12 rings, they show that the electron counts of in-plane and out-of-plane π systems can be independently modified, leading to distinct combinations of aromatic, anti-aromatic or non-aromatic behavior. This work extends the concept of aromaticity into a controllable parameter for designing novel carbon-based electronic structures.

For the Reviews, Wang and Berndt summarize the use of scanning probe techniques to investigate molecular magnetism on surfaces [7]. They highlight how methods such as scanning tunneling spectroscopy, inelastic electron tunneling spectroscopy and spin-polarized measurements enable direct access to spin excitations, magnetic anisotropy and Kondo physics at the single-molecule level. The review also emphasizes the critical role of substrate interactions in shaping magnetic behavior.

Lu provides a comprehensive overview of π-magnetism in graphene nanostructures [8]. The review outlines how sub-lattice imbalance, topological frustration and electron correlation lead to the emergence of magnetic states in nanographenes. It further discusses recent progress in on-surface synthesis and atomic-scale characterization, as well as strategies for designing magnetic graphene-based systems with tailored spin properties.

Taken together, these contributions illustrate a unifying paradigm—π-electron systems can serve as programmable quantum matter, where correlation, topology, magnetism and aromaticity are no longer fixed properties but tunable design parameters. The ability to engineer these features through chemical synthesis and surface assembly opens new opportunities for exploring fundamental quantum phenomena.

Looking forward, key challenges include achieving robust quantum coherence at higher temperatures, mitigating substrate-induced perturbations and realizing controlled coupling between multiple quantum units. Addressing these issues will be essential for translating molecular quantum systems from model platforms into functional devices. Nevertheless, the works presented here clearly demonstrate that chemistry is becoming a powerful driving force in the design of next-generation quantum materials.
